# Does learning from mistakes have to be painful? Analysis of 5 years’ experience from the Leeds radiology educational cases meetings identifies common repetitive reporting errors and suggests acknowledging and celebrating excellence (ACE) as a more positive way of teaching the same lessons

**DOI:** 10.1186/s13244-019-0751-5

**Published:** 2019-07-17

**Authors:** Andrew Koo, Jonathan T. Smith

**Affiliations:** Leeds Teaching Hospitals NHS Trust, St James University Hospital, Beckett Street, Leeds, LS9 7TF UK

**Keywords:** Learning, Errors, Discrepancies, Constructive feedback, Medical education

## Abstract

**Background:**

The Royal College of Radiologists (RCR) and General Medical Council (GMC) encourage learning from mistakes. But negative feedback can be a demoralising process with adverse implications for staff morale, clinical engagement, team working and perhaps even patient outcomes. We first reviewed the literature regarding positive feedback and teamworking. We wanted to see if we could reconcile our guidance to review and learn from mistakes with evidence that positive interactions had a better effect on teamworking and outcomes than negative interactions. We then aimed to review and categorise the over 600 (mainly discrepancy) cases discussed in our educational cases meeting into educational ‘themes’. Finally, we explored whether we could use these educational themes to deliver the same teaching points in a more positive way.

**Methods and results:**

The attendance records, programmes and educational cases from 30 consecutive bimonthly meetings between 2011 and 2017 were prospectively collated and retrospectively analysed. Six hundred and thirty-two cases were collated over the study period where 76% of the cases submitted were discrepancies, or perceived errors. Eight percent were ‘good spots’ where examples of good calls, excellent reporting, exemplary practice or subtle findings that were successfully reported. Eight percent were educational cases in which no mistake had been made. The remaining 7% included procedural complications or system errors.

**Conclusion:**

By analysing the pattern of discrepancies in a department and delivering the teaching in a less negative way, the ‘lead’ of clinical errors can be turned in to the ‘gold’ of useful educational tools. Interrogating the whole database periodically can enable a more constructive, wider view of the meeting itself, highlight recurrent deficiencies in practice, and point to where the need for continuing medical training is greatest. Three ways in which our department have utilised this material are outlined: the use of ‘good spots’, arrangement of targeted teaching and production of specialist educational material. These techniques can all contribute to a more positive learning experience with the emphasis on acknowledging and celebrating excellence (ACE).

## Key points

- Guidelines suggest that consultants should engage in and learn from discrepancy meetings.
- Positive feedback is more effective in team building than negative feedback.
- Data collated from our educational cases meeting helped to provide useful information about the pattern of recurrent discrepancies.
- The development of common recurring themes allowed relevant targeted teaching locally and nationally and production of educational material.
- Introduction of the ACE initiative encourages “good spots” to illustrate educational themes.

## Background

Most clinical departments of all specialities have a regular meeting where mistakes made are examined. The Royal College of Radiology (RCR) guidance [1] suggests that all Radiology consultants should engage in and learn from discrepancy meetings, and the General Medical Council (GMC) appraisal and revalidation guidelines all support reflection and learning from errors [2]. The estimated error rate per radiologist ranges between 3 and 5% for daily reporting with an up to 30% error rate in some retrospective studies [3–5].

Clinicians who regularly attend their departmental Discrepancy and Errors (or, in other specialities, Morbidity and Mortality) meetings are familiar with the feelings evoked when a mistake one has made arises for discussion in front of a collection of one’s peers. Negative feedback may give rise to defensiveness, shame, anger, embarrassment, insecurity and disengagement [6]. Literature from the world of educational psychology and team working in large institutions has suggested that feedback has a positive effect on clinicians’ performance [7]. Positive feedback is more effective in team building than negative feedback, and should account for more than 95% of total feedback [8]. Business teams that interact positively perform better than other teams [9]. Positive reinforcement leads to better staff engagement [8], higher morale and better team working [8]. Good team working in hospitals has been shown to improve staff performance [10], reduce stress [11] and to improve clinical outcomes [12, 13] and reduce patient mortality [14].

The questions we chose to look at were as follows: firstly, how can we reconcile the research evidence that negative feedback can be destructive to team working with the drivers to repeatedly discuss errors and discrepancies made by radiologists (for the purposes of this paper, the term ‘radiologist’ may include non-consultant reporters such as trainees, ultrasonographers and radiographers) in an open meeting? Is there a way of turning that leaden feeling of discussing mistakes into the golden feeling of learning from examples of excellent practice?

Secondly, could we identify educational themes in the recurrent errors by analysing our cases? We felt that the same mistakes were being presented over and over again, and themes were developing which, if identified, could be useful in informing future educational strategies. We wanted to take the educational cases meeting to the next stage; not just reviewing mistakes, but using the patterns of mistakes to focus and plan our teaching programme. Our first step was to identify if there was a pattern of repetitive errors which could be classified into educational themes.

Thirdly, how could we use this information to develop more positive ways of learning? We reviewed the literature on education and team working with respect to learning from errors. This led us to investigating whether these errors could be addressed in a more systematic and positive way than simply an anecdotal review of discrepancies as they arose. In our discussion, we examine ways that this information and experience could suggest other strategies including ‘good spots’, targeted teaching and development of specialist educational materials to help minimise the occurrence of the commonest errors. We hoped to provide a model that could be applied to any morbidity, mortality, errors or discrepancy meeting.

## Methods

Thirty consecutive educational cases meetings were held between 2011 and 2017 in the Department of Radiology at Leeds Teaching Hospital Trust (LTHT). LTHT is one of the largest Hospital Trusts in the UK providing imaging for one of the largest Cancer Centres in Europe. This meeting had started out as an ‘errors’ or ‘discrepancies’ meeting looking at anecdotal cases where mistakes in radiology reporting had been made. It was a poorly attended and sporadic meeting, and there was a culture of blame on the part of the radiologists discussing the cases and guilt on the part of the radiologists who had made the ‘error’. There was no evidence that practice was improving as a result, and it seemed as though similar mistakes were being made and discussed repeatedly. The way in which the meeting was rebranded has been published elsewhere [15], but during the 5 years referenced in this paper, several changes were made: the cases were anonymised; the emphasis was shifted to education not blame; feedback was constructive; attendance and engagement was linked to appraisal and revalidation; elective sessions were cancelled to allow attendance; non-medical staff, managers and trainees were encouraged to attend; mandatory training, audit presentation, focused teaching sessions and external speakers were brought in and a good lunch was served.

Complete anonymity of the patients and reporters involved in the cases has been addressed elsewhere [1, 16]. It is regarded by the authors as essential for the protection of the participants. In our Trust, an absolute division existed between the educational cases meeting and any (necessary but separate) complaints, disciplinary, investigative or legal processes which arose from errors made in radiology reporting in our department. The outcome of such formal processes may be to refer a case to the educational cases meeting, perhaps at the behest of the patients, to be anonymised and discussed to learn lessons. The educational cases meeting, however, could not in return feed into any complaints, disciplinary, investigative or legal processes. Case details and the outcome of our discussions were never made available to the Trust for such purposes. This was ensured by the chair making the cases discussed, the reporters and the case-notifiers anonymous and non-identifiable.

At the end of this 5-year period, we had accumulated a database of educational cases, mostly discrepancies that was larger than any published in the UK, and several times larger than the Royal College National Radiology Errors and Discrepancies (READ) database to whom we had contributed a large number of cases. We decided to analyse the hundreds of cases discussed and review what could be learned.

The attendance records, programmes and educational cases from 30 consecutive bimonthly meetings between 2011 and 2017 were prospectively collated and retrospectively analysed. The cases had been submitted by nearly every consultant member of the radiology department, and represented a selective, biased sample of a fraction of the number of discrepancies a department of this size is expected to have made [3]. The types of cases were determined retrospectively and divided into themes.

Please note: because of a postponed meeting in late 2015, the meeting held in January 2016 is included in the 2015 meeting data and the February 2017 meeting included in the 2016 data for ease of year-on-year comparisons.

## Results

The results are summarised in the tables below and discussed in the next section. To achieve some measure of clinical engagement and case submission (see Table 1), we looked at the number of consultants attending the meeting, how many consultants attended three meetings per year and how many consultants submitted at least one case per year, and how many sent in their own errors. We also looked at the number of ‘good spots’ which were presented, where a case which demonstrated excellent reporting rather than a discrepancy or error was submitted for discussion.

**Table 1:** Clinical engagement and case submission

	2012	2013	2014	2015	2016
Mean consultant radiologist attendance/meeting	28	29	27	31	28
% consultants attending a minimum of three meetings/year	71%	76%	65%	68%	65%
% consultants volunteering a minimum of one case/year	45%	64%	78%	88%	72%
% consultants who send in a personal discrepancy, i.e. one they reported	22%	39%	29%	23%	28%
Number of good spot case presentations	0	0	7	17	27

We then looked at the cases which had been submitted and attempted to divide them into educational themes. There were 628 cases identified from the meeting records, of which 11 were duplications or unidentifiable radiology slides with no useful supporting data. Of the 617 remaining cases, 15 filled the criteria for two of the educational themes, the rest for just one. There were, therefore, 632 cases for which an educational theme could be identified (Table 2).

**Table 2:** Themes identified from submitted cases

	Identified educational themes No of cases (percentage of total 632 cases to the nearest %)	Type of error	Number of cases
	True discrepancies		
1	Missed cancer 119 (19%)	Missed lung cancers	58
		Other missed cancers	64
2	Incorrect staging 104 (16%)	Incorrect T staging	10
		Incorrect nodal staging	18
		Incorrect staging of metastases	76
3	Misreporting of cancer 62 (10%)	Benign called cancer	40
		Cancer called benign	22
4	Fractures 36 (6%)	Missed fractures or dislocations	31
		Fracture mimics called fractures	5
5	Other clinically significant errors 161 (25%)	Non-cancer non-fracture errors incidental to reason for request (e.g. PE missed on staging CT)	36
		Non-cancer non-fracture errors relevant to reason for request (e.g. perforation of gall bladder missed on cholecystitis CT)	125
	‘Good spots’		
6	‘Good spot’ 49 (8%)	No error. Example of good practice.	49
	Other cases		
7	System error 37 (6%)	Technical/communication/protocolling/IT/delayed report errors	37
8	Educational case 53 (8%)	No error. Normal/interesting cases presented for education only	53
9	Procedural complications 8 (1%)	Complications arising from radiological procedures	8

We further analysed the cases in terms of their modality (see Table 3) and subspeciality relevance (see Table 4).

**Table 3:** Modalities of cases discussed in the educational cases meeting

Modalities	Percentage
CT	50.3%
Plain films	31.6%
MRI	7.0%
Ultrasound	6.7%
Nuclear medicine	3.9%
Fluoroscopy	0.5%

**Table 4:** Sub-speciality of the cases discussed in the educational cases meeting

Sub-specialty of the cases	Percentage of cases
Chest	27.3%
Gastrointestinal	20.7%
Genitourinary	17.2%
Musculoskeletal	16.7%
Neurology	6.3%
Vascular	5.6%
Breast	2.4%
Paediatric	2.3%
Head and neck	1.0%
Melanoma	0.5%

## Discussion

### Clinical engagement and case submission

Prior to November 2011, the radiology discrepancy meetings were attended by fewer than a dozen radiologists and approximately 20 cases were discussed per year.

After the relaunch and rebranding of the meeting in 2011, clinical engagement increased and was sustained throughout the 5-year period of the study (Table 1). Initial attendance of consultant radiologists was about 40–50% of the total consultant body per meeting (27–31/62–64) and this was maintained throughout the 5-year period, with 65–76% of consultants attending the RCR recommended minimum of three meetings per year. The proportion of consultants submitting a minimum of one case per year for discussion increased from 45 to 72% during the 5 years with a peak of 88% in 2015. Around a quarter of consultants per year sent in examples of their own mistakes, and this did not significantly change over the 5-year period (22% in 2012, 28% in 2016).

Ninety-eight percent of consultants submitted at least one case over the 5-year period. Non-consultant staff also contributed some cases. This meant that all sub-specialities were represented during the study period. Every consultant in the department attended the meeting at some time during the period of study.

Most of the cases submitted were discrepancies, or perceived errors. Some were educational cases in which no mistake had been made. ‘Good spots’ were examples of good calls, excellent reporting, exemplary practice or subtle findings that were successfully reported. The number of ‘good spots’ that were submitted annually during the 5-year period increased from 0 to 33/year. This reflected a change in the approach of the meeting to celebrate excellence as well as to discuss mistakes.

### Case analysis

Eight (1%) of the cases discussed were related to a procedural complication. Six of these were serious untoward incidents and separately investigated, two were ‘never events’ (defined as a serious incident or error that should not occur if proper safety procedures are followed). Five of these had another related educational theme, often a system or technical error (see Table 2).

Fifty-three (8%) cases were purely teaching cases in which no error had been made. Some were cases discussed in the context of targeted teaching sessions by invited speakers, some were good examples of rare findings used to illustrate a discussion of a discrepancy case and several were normal films demonstrating a particular view, technique or anatomical feature.

In 37 (6%) cases, the error was not one of interpretation by the radiologist, but a typographical, communication, systems or reporting error which had led to a clinical issue. This issue has been recognised and discussed elsewhere [16].

The cases were highly selected and from a tertiary referral centre. As such we looked at whether some modalities or sub-specialities might be under-represented (Tables 3 and 4). For example, from these tables, we can see that ultrasound was comparatively under-represented when compared to computed tomography (CT). In addition, the specialty interest of the chair (melanoma) was over-represented. This information alerted us to bias and allowed us to modify future programmes.

### True reporting discrepancies

Four hundred and eighty-five (77%) of the cases discussed in the educational cases meeting were traditional discrepancy or potential ‘errors’ cases. In these cases, the original report had a discrepancy when compared with a subsequent viewing, subsequent scan or subsequent clinical finding. Of these ‘true reporting discrepancies’, five recurrent themes were identified.

One hundred and twenty-two (25%) of the 485 discrepancies discussed were missed cancers of which almost half (*n* = 58) were missed lung lesions that were either subsequently proven cancers or had sufficient radiological features of lung cancer to necessitate further imaging. The remaining 64 were other cancers (not lung) which had been missed on initial reporting.

One hundred and four (21%) of the errors discussed were incorrect staging or restaging of cancers, most commonly missing metastases (*n* = 76) or nodal disease (*n* = 18) but also missing or mischaracterising primary recurrence (*n* = 10).

In 62 (13%) of the errors, there was an error in cancer diagnosis with either a cancer finding reported as benign (*n* = 40) or a benign finding erroneously reported as a cancer (*n* = 22).

Thirty-six (7%) were errors in fracture reporting; either missed fractures or dislocations (*n* = 31) or false positive fracture mimics (*n* = 5).

Of the 161 (33%) remaining clinically significant non-cancer, non-fracture errors, 36 cases were missed incidental signs and 125 cases were missed signs that were relevant to the referral question.

### ‘Good spots’

Forty-nine (8%) of the cases discussed in the educational cases meeting were ‘good spots’. This was an initiative introduced during the 5-year study period, where radiologists were invited to submit not only errors, but also ‘near misses’ or difficult cases in which disaster had been averted and a sharp eye had picked up a subtle finding which had been correctly reported. Often these cases illustrated the same educational points that the discrepancy cases had highlighted, but the response by the radiologists was markedly different. In each case, the pitfall which had been avoided was discussed and the methods by which the finding had been identified were held up as good practice worth aiming for. These cases were not anonymised; the reporting radiologist or radiographer responsible for the ‘good spot’ was named at the meeting, acknowledged for clinical excellence and presented with a bottle of Yorkshire Craft beer or other suitable token after discussing the case.

**Fig. 1:**
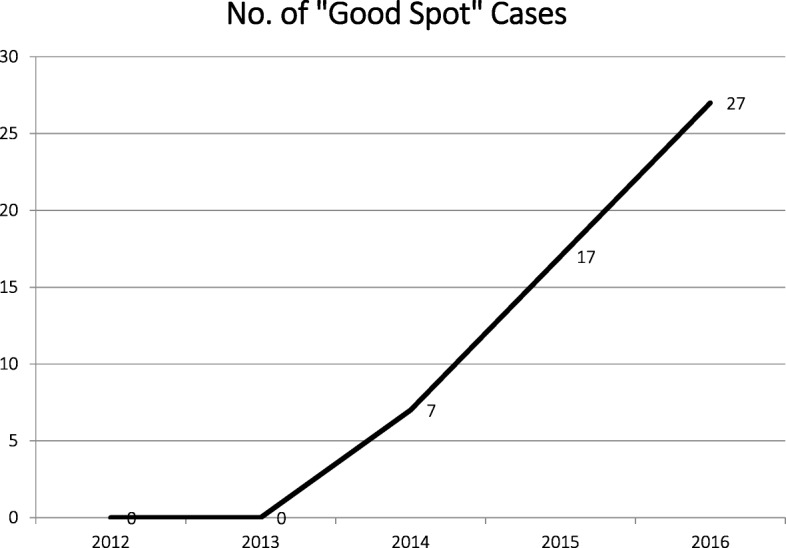
Number of ‘good spots’ each year discussed over 5 years

One of the notable results (see Fig. 1) is the increase in the use of ‘good spots’ over the 5-year study period. This was despite the overall number of cases discussed remaining fairly stable, meaning that discrepancy cases were being replaced by ‘good spots’ as the culture of the meeting changed with time.

### Using themes to improve the meeting and inform future learning

We have found that collating the data from our educational cases meeting has provided information which was useful not only to our department but also more widely. Although there is no doubt a selection bias, interrogating this in itself is useful as we can identify which modalities (such as US—see Table 3) and specialities (such as paediatrics—see Table 4) were under-represented in past meetings and adjust future programs to be more inclusive of these if necessary.

We are by no means the first to try to make sense of a database of recurrent mistakes in radiology reporting. Common misdiagnoses have previously been reported in the literature [17–19] and different approaches have been used, all with their advantages. Some publications have analysed their discrepancies anatomically [20] or arranged them by system or pathophysiology [21]; this could be useful in organ-specific targeted teaching. Others have looked at the system and organisational problems which contribute to errors such as long shifts or many consecutive days of working [22]. This may lead to discussions with the hospital Trust on how limiting shifts or encouraging breaks might decrease errors. Some have even categorised the errors on the basis of different characteristics of the radiologists [23] such as years of experience and volume of workload [24]. Morgan et al. in Leicester chose to look at a specific topic (cancer surveillance CT) and pull out the errors observed to try to learn from the commonest pitfalls [25].

Unsurprisingly, the patterns of errors we discovered in our database had many similarities to these previous studies. What we hoped to do differently was to find ways of putting this information to practical use in order to enhance the learning in our department. We did this in three ways.

Firstly, we introduced the ACE programme; acknowledging and celebrating excellence by the use of ‘good spots’ to illustrate educational themes instead of discrepancies [17]. Secondly, we organised targeted teaching on common pitfalls by experts not only within the local meeting but in a regular National Errors course which we developed and ran successfully. Thirdly, the database was used as a resource for producing educational material. Using subgroup analysis, common mistakes in any particular field, modality or sub-speciality can be targeted, as Morgan et al. demonstrated [25]. We have used our errors to produce training material for melanoma CT [26] and are in the process of producing a similar review for prostate and bladder cancer.

### The use of ‘good spots’—the ACE programme

The educational cases meeting is an opportunity for engendering good team working. West defines a functional team as having three domains; common objectives, regular meetings and interdependence [8]. A good educational meeting facilitates all of these and can have a positive impact on team morale. In fact even poor team meetings have been shown to be better than no team meetings to increase engagement and effectiveness in the NHS [10, 27].

Research supporting positive teaching is well established in educational literature [6–9, 28]. It has also been shown that positive feedback is more effective than negative feedback in corporate settings [9]. The more positive interactions a team has, the better the effect on morale and the more successful the team becomes [29]. Conversely, revisiting failure and focusing on weaknesses destroys morale and can lead to poorer team engagement [28] and distress which leads to workplace failures [30].

If regular meetings and positive feedback can improve team working, this may have an effect on patient outcomes. Work done on 1.4 million NHS workers in the UK conclusively demonstrated that the existence of effective teams had an effect on staff absence, bullying and harassment, iatrogenic staff injuries (such as needle sticks) and patient mortality. In fact, in a remarkable conclusion, a 5% increase in the number of functional teams was estimated to lead to a 3% decrease in patient mortality [31, 32].

This research made us re-evaluate the way we used errors for education in our department. It appeared that learning from mistakes was not just painful, it could be destructive. Could we use the information about the errors that were being made and deliver the educational message in a more positive way?

This led to the acknowledging and celebrating excellence (ACE) programme, where the department were asked to submit ‘good spots’ which were then used to deliver the educational lessons that the errors had previously been used to demonstrate.

The positivity of the ‘good spots’ is helpful to enable radiologists to feel safe and for trust to flourish. The feeling of safety and trust has been shown to be essential to effective team working, and to encourage innovation, progress and development in a group where these brave initiatives may have been quashed by negativity [8]. Previously, we had discussed all the errors in strict anonymity in order to make the radiologists feel safe. But we felt that a radiologist must feel safer and more celebrated within the team if s/he is being awarded a bottle of beer for a ‘good spot’ rather than watching the department discuss a mistake s/he made last month.

Below are two case examples of ‘good spots’ which we identified during educational cases meetings. The ACE initiative was used to demonstrate the exact same teaching points that recurrent errors had previously demonstrated, but with the positive effects of celebrating excellence rather than the negative effect of criticism.

**Fig. 2:**
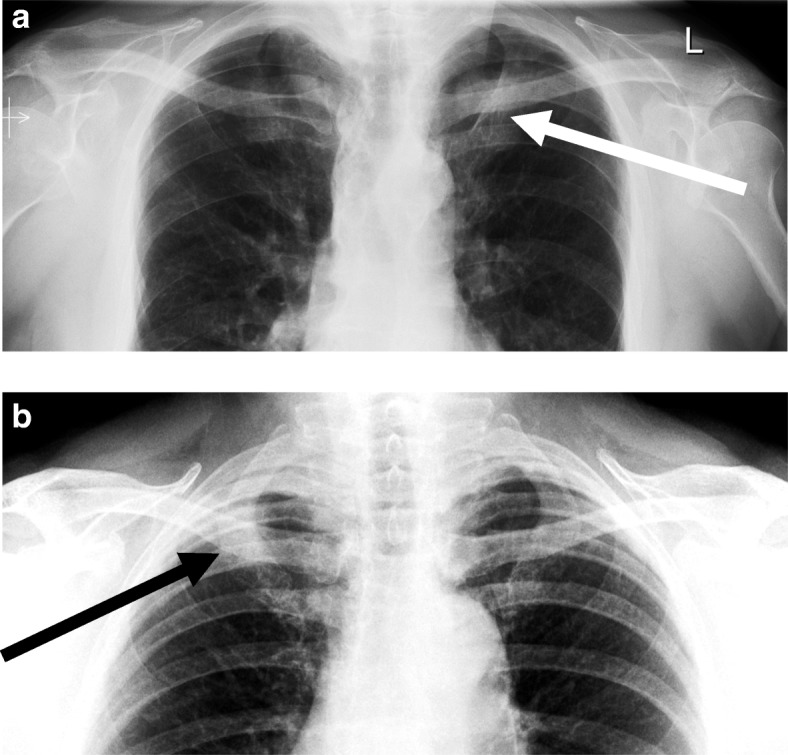
**a** Missed upper lobe lung cancer (case 535). **b** ‘Good spot’ upper lobe lung cancer (case 576a)

This patient with clinical information ‘cough’ had a chest X-ray (CXR) seen in Fig. 2a which was reported as normal. On follow up CXR and subsequent CT, a 3 cm left apical node-negative cancer was diagnosed which on retrospect was visible on the first CXR. The error was discussed in the educational cases meeting anonymously and two teaching points were emphasised. Firstly, the upper lobes are a review area. Secondly, the lung apices are difficult to interrogate due to overlapping structures therefore asymmetrical opacification should be specifically looked for and if in doubt further views or imaging sought.

In Fig. 2b, another patient with clinical information ‘1 month right upper chest pain’ had a CXR which was reported as ‘query mass right upper lobe’ and a CT was requested which confirmed the diagnosis of a T3 N0 M0 lung cancer. This ‘good spot’ was discussed in the educational cases meeting as part of the ACE initiative and the reporter was identified, congratulated and awarded the traditional bottle of Yorkshire craft beer during the meeting. The educational points which were emphasised were the same as in Fig. 2a; the upper lobes were a review area and the lung apices are difficult and further imaging (in this case CT) should be sought. In addition, it was noted that the precise clinical information on the request card was probably useful. This case delivered the same teaching points as the discrepancy case (see Fig. 2a) but with more positive feedback and an emphasis on learning from good practice rather than revisiting mistakes.

**Fig. 3:**
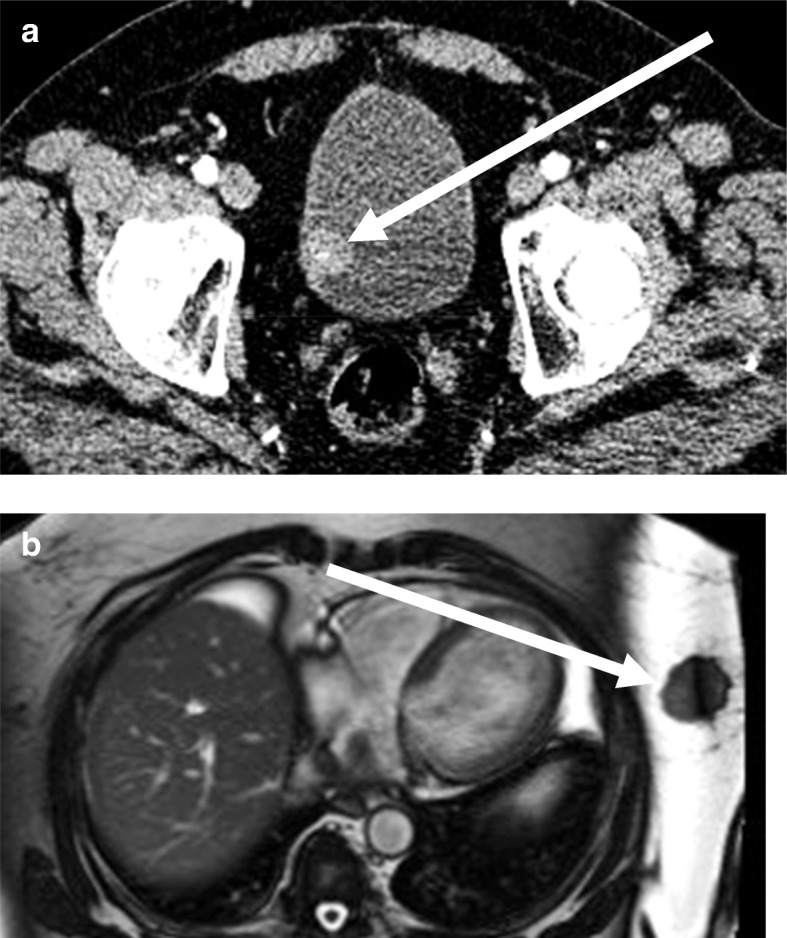
**a** Missed incidental cancer on CT (case 13, Nov 2012). **b** ‘Good spot’ incidental cancer on MR

This incidental bladder TCC seen in Fig. 3a was missed twice on subsequent CT angiograms for complex arterial disease. It was discussed as an anonymous discrepancy in our educational cases meeting with the following teaching points: (1) Be systematic when reviewing large datasets. (2) Beware satisfaction of search. (3) Incidental cancers and dual pathology are becoming more common in an ageing population.

The incidental breast cancer in Fig. 3b was picked up by one of the GI radiologists doing an MRCP for query common bile duct stones. It was discussed in our educational cases meeting as an ACE initiative ‘good spot’, and the radiologist was identified and rewarded at the meeting. The same three educational points were discussed as in 3a above, but without the associated embarrassment and with an increase rather than a decrease in morale.

### Targeted teaching by experts

Although most of us think we are getting better with experience, research has shown that clinical accuracy decreases with time unless there is focused reinforcement of learning [33–36]. The most effective educational interventions are those with an interactive component; role-play, discussion groups, case solving, etc. [37]. Continuing medical education has well-recognised benefits and is encouraged by the GMC [2] and the Royal Colleges [38].

**Fig. 4:**
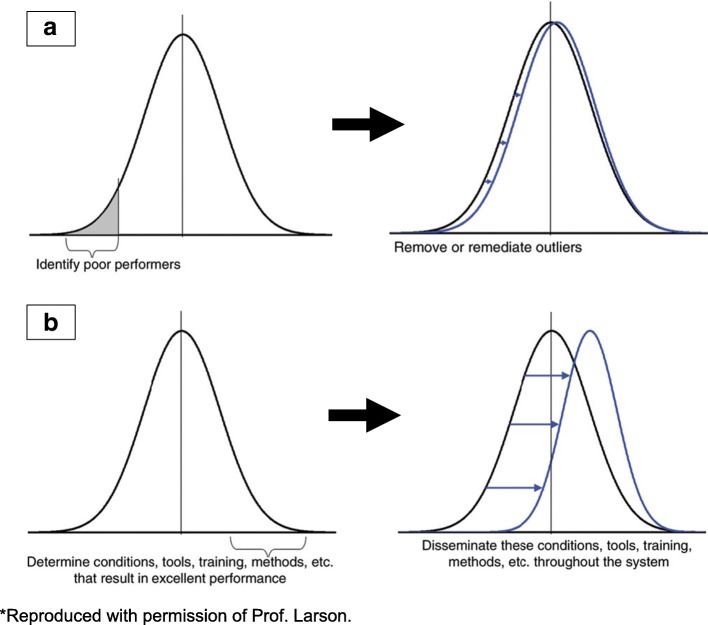
The Larson theory described that addressing poor performance (**a**) has less effect on overall results in comparison to emulating excellent performance (**b**) [39]

The identification of the common themes in our database of discrepancies allowed us to identify which areas needed targeted teaching by identifying where the recurrent mistakes were being made. We responded to this by organising targeted teaching sessions by experts to be delivered during the meetings. This would be a less negative way to emphasise an educational theme than repeatedly looking at the mistakes. Experts in the field of chest radiology, for example, could explain how they avoid the common pitfalls and demonstrate their exemplary approach to reading chest X-rays. This approach puts into practice the theory that ‘pulling’ people towards best practice is more effective than ‘pushing’ them away from poor practice as described in Larson et al. as shown in Fig. 4 [39].

By using examples of best practice delivered by experts in each field rather than looking at examples of poor practice, the same educational points can be addressed in a more constructive way, and members of the team who excel in certain areas can be used to raise the performance of their colleagues. This dissemination of learning between peers is very effective in improving team working [40].

**Fig. 5:**
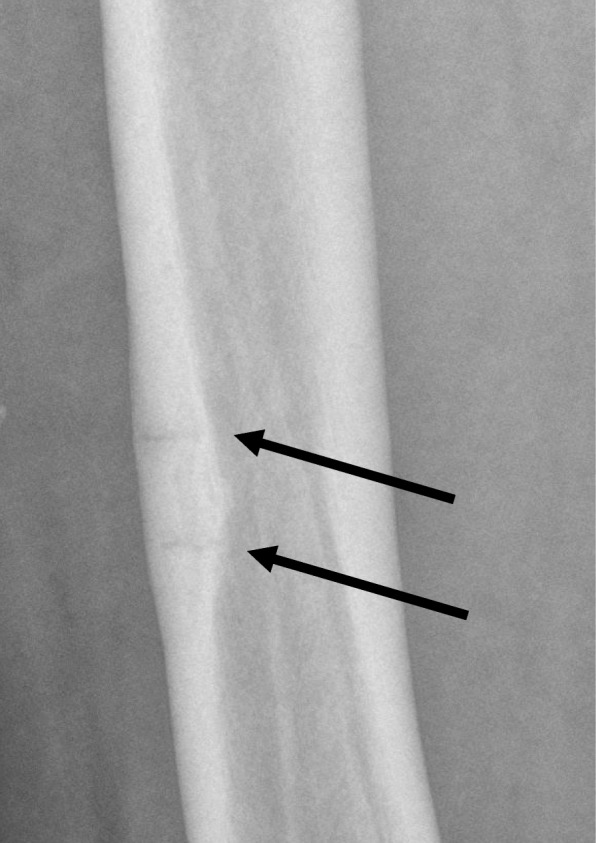
Bisphosphonate insufficiency fractures seen on a plain film of the femur

**Fig. 6:**
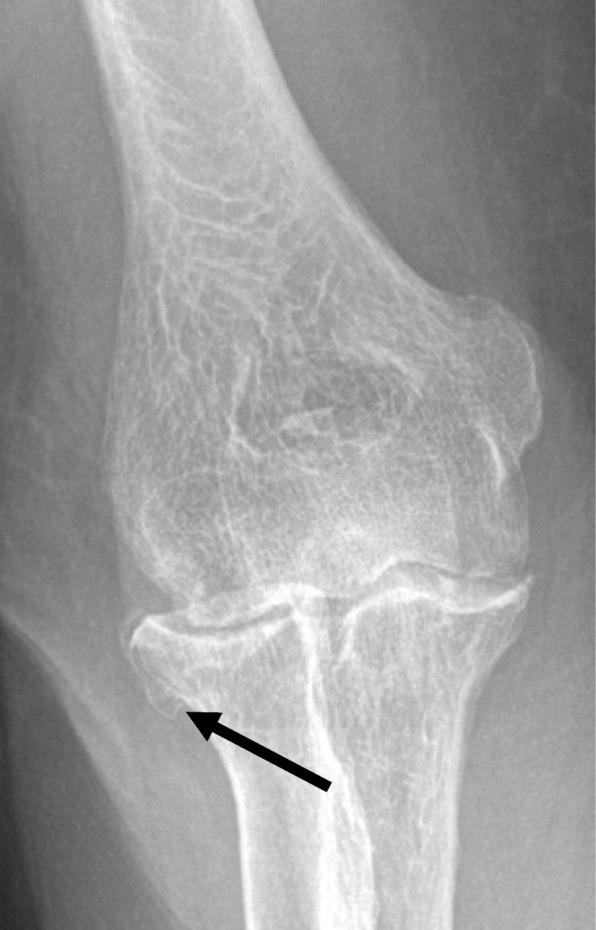
Osteophyte of the radial head mimicking an old fracture

An example of this is when we asked the musculoskeletal (MSK) team (who had been under-represented in our cases as demonstrated in Table 4) to present some cases of fracture pitfalls to the general radiology audience at the educational cases meeting. This was because fracture mimics and other pitfalls had been identified as a common recurrent error. One of these (see Fig. 5) was the underappreciated phenomenon of bisphosphonate insufficiency fracture. Another was osteophytes mimicking old fractures (see Fig. 6). Inviting experts to present educational cases like this enabled them to be discussed in a non-judgemental way with top tips on avoiding pitfalls. The experts were able to ‘pull’ the learning curve towards excellence using inspirational best practice rather than to ‘push’ the learning curve away from poor practice using fear.

**Fig. 7:**
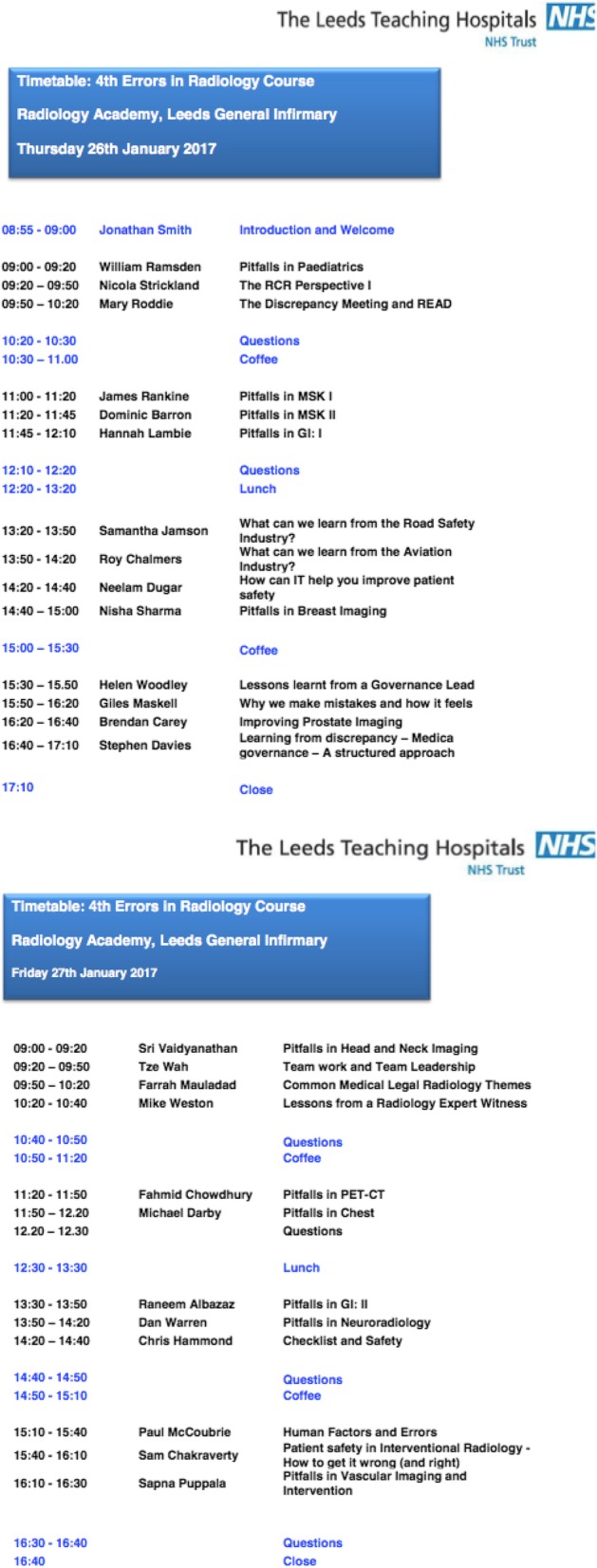
Programme for the 4th Leeds Errors in Radiology course

The success of these targeted seminars led to the National Errors course in Leeds, which has been running every other year for 8 years now. It has been oversubscribed with radiologists and trainees from throughout the UK attending, and has had universally positive feedback, with 100% of attendees saying they would recommend this course to a colleague. The 2017 meeting was expanded to a 2-day programme due to high demand, and was attended by the READ president and the incoming and outgoing presidents of the Royal College of Radiologists. Our programme consisted of general lectures on the theory of errors, and guest speakers from the aviation authority and the legal profession (see example programme Fig. 7). This was combined with clinical sessions looking at the common pitfalls in several sub-specialities delivered by experts in their field. Thus the themes identified from our educational cases meeting were utilised to select targeted teaching topics not only within our own department, but in a successful national meeting benefitting colleagues working elsewhere.

### Producing educational material

**Fig. 8:**
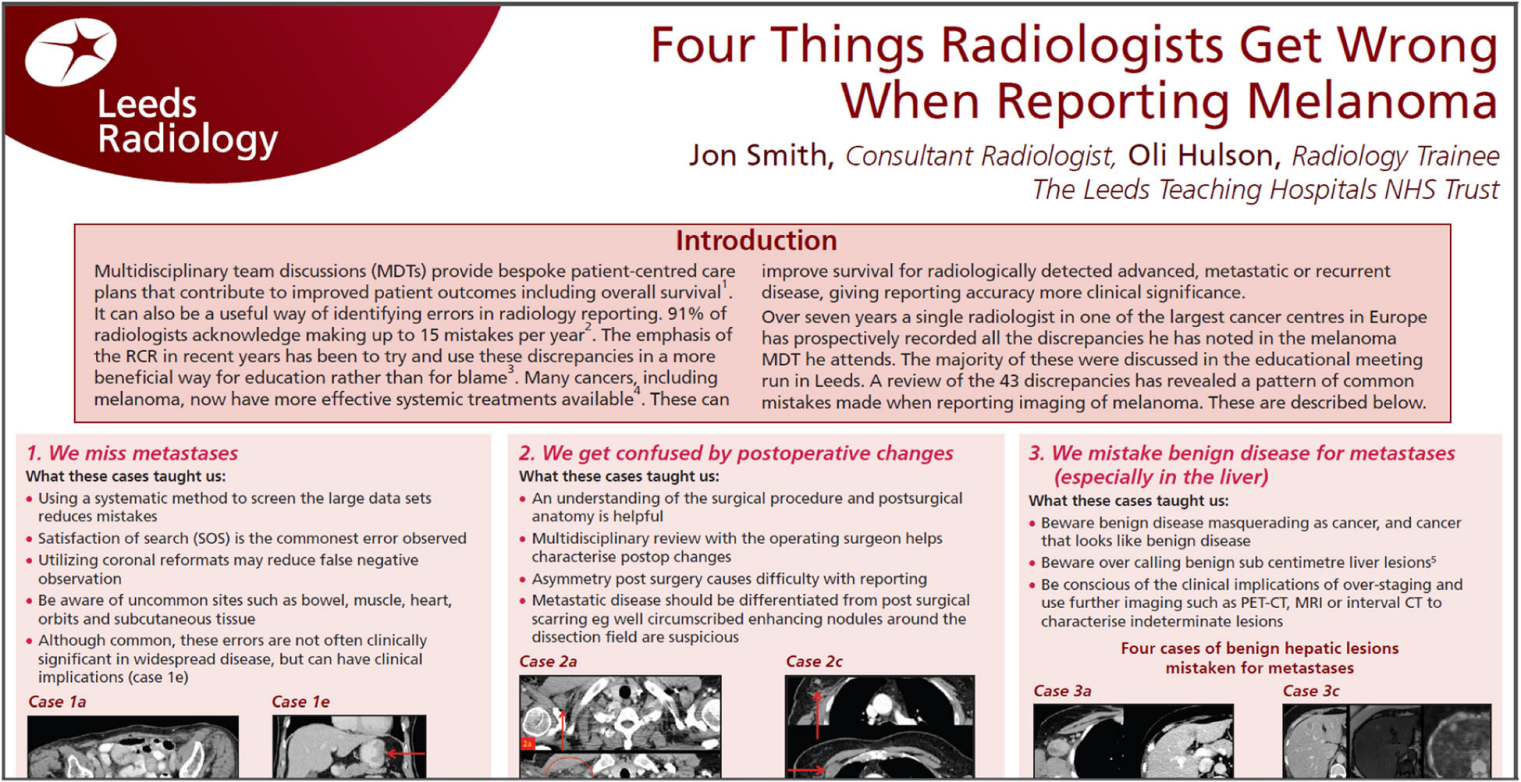
Educational material produced for melanoma

The third and final way in which the database of errors can be used to facilitate learning is in the production of educational material. Subgroup analysis of the data can provide information for specialities who wish to focus on one anatomical area, imaging modality or disease process. We have produced an educational poster entitled ‘Four things radiologists get wrong when reporting melanoma’ [26], (see Fig. 8). By looking at the errors produced over the years in melanoma CT reporting, it was possible to summarise the common pitfalls and use the educational cases to illustrate this. We are currently developing an educational interactive video and checklist along the same lines and are hoping to embed this into the CRIS system so that it is available to anyone, consultant or trainee, who is reporting a CT scan for staging or restaging melanoma. This way pertinent relevant training at the point of maximum efficacy can be delivered to prompt the reporter not to forget the common mistakes, which have been made in this area. The possibilities are endless, and two teams are currently interrogating the dataset to produce similar educational materials for bladder cancer and prostate cancer CT reporting.

## Conclusion

### Turning lead into gold

The RCR and GMC encourage learning from mistakes, and most radiology departments have meetings to look at their errors. But this can be a demoralising process with negative implications for staff morale, clinical engagement, team working and patient outcomes. By analysing the pattern of discrepancies in a department and delivering the teaching in a less negative way, the ‘lead’ of clinical errors can be turned in to the ‘gold’ of useful educational tools. Interrogating the whole database periodically can enable a more constructive, wider view of the meeting itself, identify recurrent deficiencies in practice and point to where the need for continuing medical training is greatest. A regular, non-judgemental, anonymous, inclusive educational cases meeting is vital. The use of ‘good spots’, targeted teaching and specialist educational material can all contribute to a more positive learning experience with the emphasis on acknowledging and celebrating excellence (ACE).

## Abbreviations

ACE: Acknowledging and celebrating excellence
CT: Computed tomography
CXR: Chest X-ray
GMC: General Medical Council
LTHT: Leeds Teaching Hospital Trust
MSK: Musculoskeletal
RCR: Royal College of Radiologists
READ: Radiology Errors and Discrepancies Database
